# Squamomelanocytic Tumor, An Entity Still Shrouded in Mystery: Case Report and Literature Review

**DOI:** 10.3390/dermatopathology12010001

**Published:** 2025-01-13

**Authors:** Joana Sorino, Mario Della Mura, Anna Colagrande, Cecilia Salzillo, Giuseppe Ingravallo, Gerardo Cazzato

**Affiliations:** Section of Molecular Pathology, Department of Precision and Regenerative Medicine and Ionian Area (DiMePRe-J), University of Bari “Aldo Moro”, 70124 Bari, Italy; sorino.joana@hsr.it (J.S.); mario.dellamura1@gmail.com (M.D.M.); anna.colagrande@gmail.com (A.C.); ceciliasalzillo89@gmail.com (C.S.); giuseppe.ingravallo@uniba.it (G.I.)

**Keywords:** squamomelanocytic tumor, melanoma, squamous cell carcinoma, melanocarcinoma, collision, combined, PRAME

## Abstract

Cutaneous squamomelanocytic tumor (SMT) is a very rare cutaneous malignancy, composed of a dual phenotypic population of both malignant melanocytes and keratinocytes, intimately intermingled together. Herein, we report a new case of a SMT occurring in an 82-year-old man, located on the scalp. Histopathology revealed a mixed population consisting of squamous cell carcinoma and melanoma within the same lesion, also confirmed using immunohistochemical staining for high molecular-weight cytokeratins (HMWCKs) and Melan-A. Moreover, to the best of our knowledge, for the first time, we tested SMT for the preferentially expressed antigen in melanoma (PRAME), which revealed a strong and diffuse positivity in the melanocytic component. These tumors need to be distinguished by more frequent collision tumors and colonization. Furthermore, we provide a comprehensive review of the literature, focusing on clinical and histopathological aspects, biological behavior and still-debated, but fascinating histogenesis of this elusive entity.

## 1. Introduction

Squamomelanocytic tumor (SMT) is a very rare entity, representing a combined skin tumor consisting of a dual population of neoplastic melanocytes and keratinocytes intimately intermingled together. To date, only a few cases have been reported in the literature, since the first descriptions provided by Rosen et al. and Pool et al. [1,2]. These neoplasms must be differentiated from more frequent collision tumors and colonization, and their histopathogenesis is still an enigma. Their clinical behavior is probably more indolent than conventional melanoma; however, there is a general lack of definitive data about this rare and elusive entity. Herein, we report a new case of SMT occurring in an 82-year-old man. Moreover, we provide a comprehensive review of the literature, with the aim to deepen the clinicopathological features and histogenesis of these neoplasms.

## 2. Materials and Methods

The clinical history of the patient was retrieved from medical records. Tissue samples were formalin-fixed and paraffin-embedded (FFPE) and stained with hematoxylin and eosin (H&E). Immunohistochemical coloration for cytokeratin AE1-AE3 (CK-AE1/AE3), Melan-A, preferentially expressed antigen in melanoma (PRAME), BRAF V600E, p53 and p16 were also performed to further investigate neoplastic cells.

The research was carried out on PubMed, using the following words variously arranged together: “squamous cell carcinoma”, “melanoma”, “squamomelanocytic tumor”, “melanocarcinoma”, “combined”, “collision”, “biphasic”, “biphenotipic” and “coexisting”. All English-language abstracts or full articles found were evaluated and all cases of skin tumors constituted by a malignant squamous cell and a malignant melanoma component intermingled together (i.e., true SMT) were selected for the discussion.

## 3. Case Presentation

An 82-year-old Caucasian man was admitted to our hospital with a brownish skin nodule of 3.5 cm, located on the scalp. The clinical suspicion was non-melanoma skin cancer; consequently, an excisional biopsy was performed.

**Figure 1:**
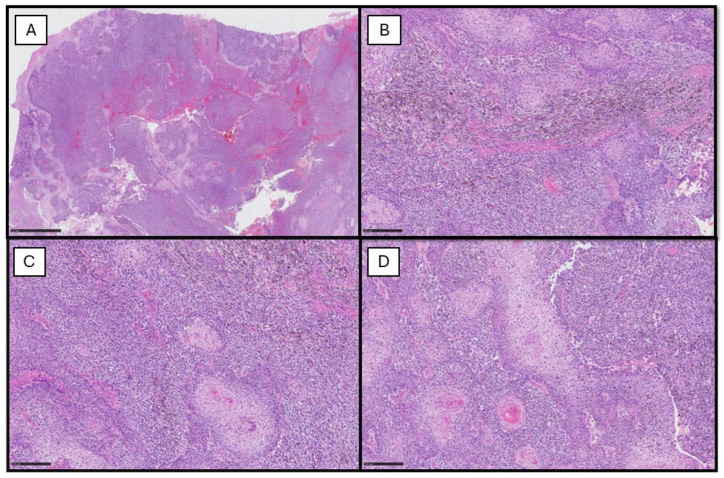
(**A**) Histopathological photomicrograph showing the neoplastic lesion constituted by two different components (hematoxylin–eosin, original magnification 4×), scale bar 1 mm. (**B**) Higher magnification showing melanocytic component with melanin pigment and squamous component (hematoxylin–eosin, Original Magnification 10×), scale bar 250 µm. (**C**,**D**) The neoplastic population is made up of sheets of melanoma cells intermingled with islands of squamous cells showing central horn pearl formations and focal clear cell changes. Melanin pigment deposition is observed throughout the lesion (hematoxylin–eosin, original magnification 10×), scale bar 250 µm.

Histopathology revealed an exophytic, extensively ulcerated tumor, diffusely infiltrating the superficial and deep dermis and associated with abundant necrosis. Cytologically, it was made up of two phenotypically distinct populations, intimately intermingled together and almost blending into each other at several points (Figure 1). The first component consisted of atypical squamous cells distributed in nests and cords, with true keratinization, dyskeratinocytes and horny pearl formation; clear cell changes were also noted. The second component consisted of epithelioid, to a lesser extent spindle-shaped, melanocytes, with markedly atypical nuclei, prominent nucleoli and eosinophilic cytoplasm. A significant melanin deposition was observed. Focal epidermal connection was observed at the edges of the lesion; however, a true melanoma in situ component was not identified. The mitotic activity was brisk in both populations (up to 15 mitoses/mm^2^), including atypical mitosis.

Breslow thickness was equal to 10 mm. Focal vascular invasion was observed. There were no brisk tumor-infiltrating lymphocytes (TILs) nor evidence of regression; by contrast, multiple plasma cell aggregates and a background of severe solar elastosis (cumulative solar damage: 3) were seen in the surrounding dermis.

**Figure 2:**
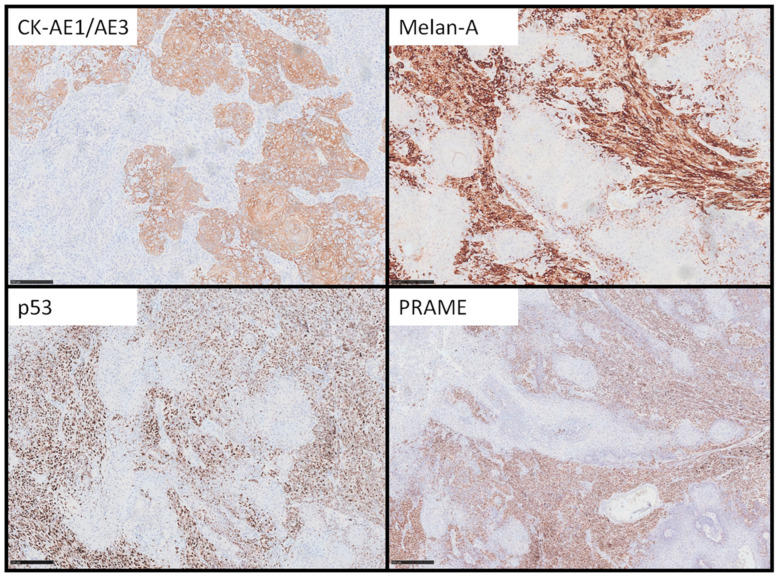
Immunohistochemistry confirmed the dual nature of neoplastic population: squamous cells were positive for CK-AE1/AE3, instead melanocytes expressed Melan-A. p53 showed strong nuclear positivity in melanocytes, suggesting an underlying mutation. Also, PRAME was strongly positive in the melanocytic component (immunohistochemistry, original magnification 10× and 5×), scale bar 250 µm (A–C) and 500 µm (D).

Immunohistochemistry showed a dichotomous pattern (Figure 2): squamous cells expressed CK-AE1/AE3 while the melanocytic component was positive for Melan-A and PRAME. p53 showed a strong nuclear expression in melanoma cells, suggesting an underlying mutation. BRAF V600E and p16 were negative in both populations. To the best of our knowledge, this is the first time SMT has been tested for PRAME.

## 4. Discussion

Skin cancers are usually classified as melanoma skin cancers, deriving from melanocytes, and non-melanoma skin cancers, usually deriving from keratinocytes. However, cutaneous neoplasms from both cell types can exist within the same lesion, which can be classified into collision tumors, combined tumors and tumors with secondary colonization.

Collision tumors are single macroscopic lesions consisting of two adjacent/juxtaposed histologically different neoplasms, each one showing clearly demarcated boundaries. They can occur with a certain frequency and are mostly represented by collisions between basal cell carcinoma (BCC) and malignant melanoma (MM) [3]; cases involving squamous cell carcinoma (SCC) and MM can be also observed; however, they should not be considered SMT but “MM associated/adjacent to SCC”.

Combined tumors are hybrid neoplasms in which the same lesion is composed of two phenotypically different, cytologically distinct neoplastic populations, intimately intermixed with one another and embedded within the same stroma. The presence of a melanomatous component is rare and occurs in true SMT, basomelanocytic tumors and trichoblastomelanoma [4]; a combined tumor consisting of keratoacanthoma and melanoma and a ‘triple combined’ baso-squamo-melanocytic tumor have also been described [3,5]. The term melanocarcinoma has also been employed for those entities, although it should be discouraged since originally employed for melanoma [6].

Colonization consists of the secondary colonization of an epithelial tumor (e.g., BCC, SCC) by melanocytes, which can be benign dendritic melanocytes or atypical melanocytes spreading from an adjacent melanoma in situ [7].

A total number of 44 SMTs have been reported in the English literature so far, including our case (Table 1) [1,2,4,6,7,8,9,10,11,12,13,14,15,16,17,18,19,20,21,22,23,24,25,26,27,28,29]. Affected patients are more frequently males (30 males, 14 females), with a mean age of 69.8 years (range: 32–94). The most common site of presentation is the head and neck area, while the involvement of the oral mucosa and the plantar surface is the rarest. Clinically, they usually present as brownish exophytic lesions, often crusted or ulcerated, ranging in size between 0.2 to 7 cm, although non-pigmented lesions have been described [4,8].

Histologically, they generally present as dermal-centered nodules, sometimes showing partial epidermal involvement, with a Breslow thickness ranging from <0.75 to 12 mm. The neoplastic cells show partly keratinocytic differentiation, with true keratinization, horn pearl formation and high molecular weight cytokeratin (HMWCKs) positivity, and partly melanocytic differentiation, with dendritic, epithelioid and/or spindled morphology and reactivity for melanocytic markers (e.g., S-100, Melan-A, HMB45). BRAFV600E mutation, commonly seen in melanomas arising in sun-exposed skin, was not found in our case and in the other case in which immunohistochemical evaluation was carried out [25]. True ‘biphenotipia’ has been described in four cases, consisting of co-expression of HMWCKs and melanocytic markers, or the presence of both tonofilaments with well-developed desmosomes and premelanosomes/melanosomes within the same cells [1,8,21,22]. A single case demonstrated a third cell population negative for both keratinocytic and melanocytic markers [12], probably indicating a less differentiated or more immature component. Finally, two cases showed foci of adnexal differentiation within the squamous cell areas consisting of focal matrical differentiation with ghost keratinocytes and giant cell reaction [19], or abortive ductal/glandular structures EMA + CEA^+7^, in contrast with conventional SCC.

The main differential diagnoses consist of: pseudoepitheliomatous hyperplasia accompanying MM, characterized by reactive non-neoplastic keratinocytes; collision between MM and SCC; melanocytic colonization of SCC or adnexal-derived neoplasms (e.g., melanotic matricoma and pilomatrix carcinoma), in which melanocytes are benign or result from extension of the adjacent melanoma in situ; MM with aberrant CK expression, in which true keratinization is not observed.

To date, the presence of both melanocytic and squamous cell features in a solitary cutaneous tumor still constitutes a histogenetic enigma, challenging our current knowledge about skin tumor biology. This complexity may be related to the multitude of factors contributing to tumorigenesis. The great majority of SMTs arise in the sun-exposed skin areas of old people, thus ultraviolet radiation probably represents a major etiological factor; however, it cannot explain the rare cases of SMTs arising in non-sun-exposed areas, such as the oral cavity [28] and the plantar surface [23]. Although multiple theories have been proposed to explain the development of SMTs, a definitive consensus has yet to emerge. Here, we summarize the three most widely accepted theories about the histopathogenesis of this rare, combined tumor, which are not mutually exclusive: convergent theory, divergent theory and interaction theory.

According to the convergent theory, tumor cells arise from an immature multipotent stem cell located in the epidermis or dermis, that, during the neoplastic transformation, differentiates divergently, resulting in a dual neoplastic population (i.e., keratinocytes and melanocytes) [3,22]. The presence of multipotent stem cells, capable of differentiating in multiple lineages including melanocytes, has been demonstrated in normal hair follicles as well as in the dermis of normal human foreskin, thus also explaining SMT arising in glabrous skin [30,31]. This theory is supported by the presence of SMT cases showing a ‘true biphenotipia’, as already illustrated, as well as the frequent presentation as a dermal-based nodule without an in situ component.

In contrast, the divergent theory postulates that melanoma cells can exhibit significant phenotypic plasticity, demonstrating the ability of some neoplastic subclones to transdifferentiate towards a non-melanocytic phenotype, including epithelial or adnexal cells, probably due to extracellular factors produced within the tumor microenvironment^31^, as occasionally also observed in other neuroectodermic-derived tumors like glioblastoma. However, the plausible mutation of the *TP53* in melanoma but not in squamous cell carcinoma component discourages this hypothesis, almost in our case.

Finally, the interaction theory suggests that combined tumors arise from an interplay between the two different populations, wherein the primary tumor induces the development of the second neoplastic component within the same lesion via a paracrine mechanism [4,9]. This is corroborated by the evidence that, in the normal epidermal melanin units, melanocytes and keratinocytes engage a reciprocal crosstalk, with the former releasing melanin to supply keratinocytes and the latter exerting a regulatory effect on melanocytes growth. This equilibrium can be disrupted when neoplastic keratinocytes overproduce growth factors, promoting an uncontrolled proliferation of melanocytes and ultimately leading to their neoplastic transformation.

The biological behavior of SMT is largely unknown, due to its rarity. Therefore, it is difficult to assess prognostic implications and proper therapeutic approaches. Based on data from the available literature, SMT seems to be a less aggressive neoplasm in comparison to conventional melanoma of equal Breslow thickness, exhibiting a more indolent clinical course. To date, only two patients developed micrometastases within the sentinel lymph node, in both cases consisting of a pure melanoma component [15,17], and, in only one report, the patient died of disease [28]; in all other cases, they survived without signs of recurrence, with a mean follow-up time of 29.6 months (range: 0–167). This fact may be attributable to a heightened dependence of the invasive melanoma component on the epithelium or epithelium-derived factors, which can also act as a barrier to the systemic spreading [4,22]. However, we prudentially recommend managing SMT as a conventional melanoma, given the scarcity of data and the lack of definitive guidelines.

## 5. Conclusions

Squamomelanocytic tumors represent a rare and enigmatic category of combined cutaneous neoplasms, characterized by the intermingling of melanocytic and keratinocytic populations within a single lesion. Their precise histopathogenesis remains unclear, with multiple theories, including convergent, divergent and interaction models providing plausible explanations for their development. The current case of an 82-year-old male with SMT adds to the limited body of literature, offering unique insights into the clinicopathological features of this entity, including histological findings and immunohistochemical profiles.

From a clinical perspective, SMTs typically present in sun-exposed areas, predominantly in elderly males, and display a dermal-based growth with diverse histological patterns. Despite their aggressive histological appearance, including high mitotic activity and substantial Breslow thickness, SMTs generally exhibit less aggressive clinical behavior compared to conventional melanomas. However, due to limited data and the potential risk of metastasis, especially of the melanocytic component, managing SMTs with the same caution as conventional melanoma is advisable.

Further research, including molecular and genetic studies, is essential to elucidate the biological behavior, prognostic factors and optimal management strategies for SMTs. This case highlights the importance of comprehensive histopathological and immunohistochemical evaluation in distinguishing SMTs from collision tumors and other differential diagnoses, thereby contributing to the broader understanding of this rare tumor type.

## Figures and Tables

**Table 1:** The table summarizes the 44 cases of SMT so far reported in the literature, comprehensive of our case. Reports are listed in chronological order including all available information about localization, dimension, Breslow thickness and follow-up status. The table has been adapted from Lòpez-Llunell et al. [27].

No.	Authors	Year of Publication	Age/Sex	Localization	Tumour Dimension (cm)	Breslow (mm)	Sentinel Node	Follow-Up (Months)
1	Rosen et al. [1]	1984	-	Face	-	-	NA	NA
2 3 4 5	Pool et al. [2]	1999	70/M 50/M 44/F 47/M	Canthus Eyebrow Forehead Nose	1 0.3 0.8 0.3	2.7 2 1.9 1	NA NA NA NA	12 24 108 12
6	Cutlan et al. [8]	2000	72/F	Shoulder	0.5	-	NA	NA
7	Ahlgrimm-Siess et al. [9]	2007	84/F	Cheek	-	<0.75	NA	NA
8	Doric et al. [10]	2008	61/M	Preauricular	1.2	-	NA	48
9	Falanga et al. [11]	2008	46/M	Lower lip	-	6	Negative	NA
10	Rongioletti et al. [7]	2009	94/M	Back	1	-	NA	8
11	Leonard et al. [12]	2009	68/M	Temple	0.4	-	NA	0
12	Pouryazdanparast et al. [13]	2009	62/M	Ear	-	2.1	Negative	9
13	Miteva et al. [4]	2009	82/F	Nose	1	In situ	NA	NA
14 15 16	Satter et al. [14]	2009	73/F 76/F 63/F	Arm Arm Leg	- - 2.5	1.9 2.7 1.6	Negative Negative Negative	27 25 31
17	Amerio et al. [15]	2011	32/F	Arm	0.8	4.3	Micrometastasis	10
18 19	Scruggs et al. [16]	2011	80/M 65/F	Temple Nose	0.7 0.5	2.04 1.7	NA Negative	12 12
20	Haenssle et al. [17]	2012	75/M	Thumbnail	-	4.2	Micrometastasis	24
21	Wong et al. [18]	2013	83/F	Temple	0.7	-	NA	36
22	Rodic et al. [19]	2013	72/M	Nose	0.2	1.2	Negative	6
23	Wang et al. [20]	2013	63/F	Canthus	1.2	12	NA	14
24	Jour et al. [21]	2014	78/M	Retroauricular	0.5	-	NA	NA
25 26 27 28 29 30 31 32 33 34 35	Amin et al. [22]	2015	86/M 68/M 71/M 87/M 81/M 53/M 80/F 46/F 75/M 72/M 52/M	Cheek Ear Nose Cheek Scalp Hand Arm Arm Scalp Arm Scalp	- - - - - - - - - - -	1.3 2.6 1.33 1.99 2.8 3.2 2.5 0.75 In situ In situ In situ	NA Negative Negative NA Negative NA NA Negative NA NA NA	25 NA 45 21 48 13 NA 42 167 NA NA
36	Kochoumian et al. [6]	2015	78/M	Forearm	-	-	NA	NA
37	Malhotra et al. [23]	2016	70/M	Foot (plantar)	1	-	NA	6
38	Mangkorntongsakul et al. [24]	2020	70/M	Scalp	3.5	7.4	NA	12
39	Diamantopoulos et al. [25]	2021	69/M	Back	-	-	Negative	NA
40	Zipperer et al. [26]	2022	87/M	Shoulder	2.5	>2	NA	12
41	Lopez-LLunell et al. [27]	2023	84/M	Mandibular angle	1	3.8	NA	15
42	Renuga et al. [28]	2024	48/F	Oral cavity	7	-	NA	DOD
43	Daruish et al. [29]	2024	94/M	Forehead	-	1.5	NA	NA
44	Present Case	2024	85/M	Scalp	3.5	10	NA	NA

NA not available or not performed, DOD died of disease.

## Data Availability

Data are contained within the article.
